# Consent to organ offers from public health service “Increased Risk” donors decreases time to transplant and waitlist mortality

**DOI:** 10.1186/s12910-022-00757-0

**Published:** 2022-03-05

**Authors:** Yvonne M. Kelly, Arya Zarinsefat, Mehdi Tavakol, Amy M. Shui, Chiung-Yu Huang, John P. Roberts

**Affiliations:** 1 Department of Surgery, University of California, 400 Parnassus Avenue, A730K, San Francisco, CA 94117 USA; 2 Department of Epidemiology and Biostatistics, University of California, San Francisco, CA USA

**Keywords:** Communication, Increased risk, Informed consent, Organ donation, Risk perception

## Abstract

**Background:**

The Public Health Service Increased Risk designation identified organ donors at increased risk of transmitting hepatitis B, hepatitis C, and human immunodeficiency virus. Despite clear data demonstrating a low absolute risk of disease transmission from these donors, patients are hesitant to consent to receiving organs from these donors. We hypothesize that patients who consent to receiving offers from these donors have decreased time to transplant and decreased waitlist mortality.

**Methods:**

We performed a single-center retrospective review of all-comers waitlisted for liver transplant from 2013 to 2019. The three competing risk events (transplant, death, and removal from transplant list) were analyzed. 1603 patients were included, of which 1244 (77.6%) consented to offers from increased risk donors.

**Results:**

Compared to those who did not consent, those who did had 2.3 times the rate of transplant (SHR 2.29, 95% CI 1.88–2.79, *p* < 0.0001), with a median time to transplant of 11 months versus 14 months (*p* < 0.0001), as well as a 44% decrease in the rate of death on the waitlist (SHR 0.56, 95% CI 0.42–0.74, *p* < 0.0001). All findings remained significant after controlling for the recipient age, race, gender, blood type, and MELD. Of those who did not consent, 63/359 (17.5%) received a transplant, all of which were from standard criteria donors, and of those who did consent, 615/1244 (49.4%) received a transplant, of which 183/615 (29.8%) were from increased risk donors.

**Conclusions:**

The findings of decreased rates of transplantation and increased risk of death on the waiting list by patients who were unwilling to accept risks of viral transmission of 1/300–1/1000 in the worst case scenarios suggests that this consent process may be harmful especially when involving “trigger” words such as HIV. The rigor of the consent process for the use of these organs was recently changed but a broader discussion about informed consent in similar situations is important.

## Background

In 1991 an intensive investigation led by the Centers for Disease Control (CDC) and Public Health Service (PHS) determined that several organ and tissue transplant recipients had contracted human immunodeficiency virus (HIV) from donors who had tested negative for the HIV antibody at the time of donation. These seminal cases of HIV transmission led to the formation of a working group which ultimately published a set of guidelines from the CDC and PHS aimed at reducing the risk of HIV transmission from organ donor to recipient in 1994 (1). These guidelines included standardized anti-HIV antibody testing of all potential organ donors as well as a comprehensive risk assessment to identify donors with designated high-risk behaviors in the 12 months to 5 years prior to donation which would put them at higher risk for contracting and thereby transmitting HIV. All donors who met the criteria outlined in these initial guidelines were labeled “PHS High Risk” and beginning in 2007 the Organ Procurement and Transplantation Network (OTPN) required transplant centers to document specific informed consent of these risks with potential organ recipients agreeing to accept organs offers from these groups. In 2013, these PHS guidelines were updated, with changes made to the definitions of high-risk behaviors, alterations in testing requirements, official incorporation of hepatitis B virus (HBV) and hepatitis C virus (HCV), and with new donor designation now being termed “PHS-Increased Risk” (PHS-IR) (2). With the opioid epidemic as well as the addition of the criteria of hemodialysis in the preceding 12 months, the percentage of organ donors with the increased risk designation increased from 9.35% in 2010 to 26.2% in 2017 (3, 4).

The inclusion of donors with these behavioral risks within the donor pool is widely accepted in order to help narrow the gap between the supply and demand of this scarce resource, however the approach to informing and consenting potential recipients of these organs has been highly variable among centers as evidenced by rates of acceptance of offers from these donors from 2 to 95% across the 58 donor service areas (5). Despite the label of “Increased Risk”, the absolute risk of disease transmission from these donors compared to standard criteria donors is low and has only decreased further in the era of standardized nucleic acid testing of organ donors. Prior to the 2013 update, recommended testing for HIV, HBV, and HCV in organ donors was via antibody or antigen testing on donor serum. While these serologic tests are generally highly sensitive, donors who become infected in close proximity to their death may not have positive antibodies or detectable circulating antigen, and therefore may have negative serologic testing and yet transmit the infection to the organ recipient, the so-called window period (6, 7). The advent of nucleic acid testing (NAT) further reduced this window period, with studies showing that the period of time between disease acquisition and positive NAT is 5–6 days for HIV, 3–5 days for HCV and 20–22 days for HBV (8, 9).

With improvements in screening and testing, studies have shown an extremely low rate of disease transmission. Studies examining HIV incidence in the various PHS-IR categories found the risk of window period infection to range from 0.04/10,000 in patients with hemophilia to 4.9/10,000 in patients using intravenous drugs, while similar studies examining HCV incidence in the various PHS-IR categories found the risk of window period infection to range between 0.03–32.4/10,000, and for HBV to range from 4.5–8.9/10,000 (7, 10, 11). A CDC modeling study found that the risk of a donor having undetected infection was less than 1/1,000,000 if the NAT remained negative for 14 days for HIV, 35 days for HBV, and 7 days for HCV after the donor’s most recent exposure (12). The increased risk designation was based upon a 12-month time frame for the behavior or circumstances, such as imprisonment, which is obviously much longer than the window periods. The CDC determined that the risk for undetected infection in donors with increased-risk behaviors screened by NAT 30 days after the most recent potential risk behavior was fewer than one per 1 million for HIV and hepatitis C and close to one per 1 million for hepatitis B (12).

Despite data showing these organs are safe for transplant, the fear of acquiring these diseases or the stigma associated with receiving organs from “increased risk” groups drove patients to decline organs offers from these groups, despite the fact that these donors were younger and overall healthier donors (13–16). This concern may also weigh on transplant professionals and contribute to underutilization of organs from increased-risk donors, which are discarded at rates up to 1.5 times that of organs from standard criteria donors (17–21).

Based upon a number of factors, PHS issued updated guidelines in 2020 that have now been adopted which move to eliminate the “increased risk” terminology from the process of discussing organ offers with potential recipients and to alter the consent process such that potential recipients are informed of a donor’s individualized risk profile at the time of organ offer without need for a separate informed consent process for donors with potentially higher risk of viral transmission (22).

For patients awaiting liver transplantation, access to offers from this substantial portion of the donor pool could have a significant effect on outcomes. We hypothesize that patients who consent to receive organ donation offers from PHS-IR designated donors will have both a decreased time to transplant and a lower waitlist mortality compared to patients who do not consent to receiving organ offers from PHS-IR donors.

## Methods

### Study population

We performed a single-center, retrospective review of all patients waitlisted for liver transplantation at the University of California, San Francisco from August 2013 to November 2019. A total of 1682 patients were listed for liver transplantation during this period. Seventy-nine records were excluded from the analysis either due to incomplete information or inaccurate information: n = 25 lost to follow up or transferred to another center, n = 22 removed from the waitlist because they could not be contacted, and n = 32 with conflicting dates (i.e. event date prior to listing date) This left 1603 unique patient records to be included in our analysis.

This study was approved by the Institutional Review Board of the University of California, San Francisco (IRB study approval number 19-27692) and all related research and methods were performed in accordance with the relevant guidelines and regulations set forth by the IRB. The review board waived the requirement for informed consent for this research and none of the patients included were from vulnerable populations.

### Informed consent

At the University of California, San Francisco from August 2013 to November 2019, an informed consent discussion was conducted with each patient and their family by the transplant hepatologists after the patient was listed for liver transplantation. During this discussion, the standard risks associated with transplant were discussed, along with the risks of viral transmission from standard and increased risk criteria donors. Several subcategories of donors, including donation after cardiac death, older donors, split liver donors, donors with history of cancer, and PHS-IR criteria donors, were discussed with each patient and their family.

### Data collection

The following recipient-related variables were obtained from the UCSF Transplant Database: age at time of listing; sex; race; blood type, height and weight at listing (from which body mass index (BMI) was calculated); etiology of liver disease; Model for End-Stage Liver Disease (MELD) and its individual components (serum bilirubin, creatinine, and INR) at time of listing; date of listing; date of transplantation; date of death; date of removal from the wait list; cause of removal from the wait list; and consent to receiving offers from PHS “increased risk” donors.

### Statistical analysis

Patient baseline characteristics were compared between consent status groups using chi-square and t-tests. The difference in time to transplant between groups was assessed using Gray’s test for equality of cumulative incidence functions. The three competing risk events (transplant, death, and removal from transplant list) were summarized using cumulative incidence functions (CIFs) by consent status. The association between consent status and the competing events was evaluated using a Fine-Gray sub-distribution hazard model with consent to organ offers from Public Health Service-Increased Risk (PHS-IR) donors as a covariate. The sub-distribution hazard of a competing event measures the risk of the competing event in participants who have not yet experienced an event of that type. To control for potential confounding effects, multivariable analyses were also performed by including recipient age at listing, race, gender, blood type, and MELD at listing as covariates in the Fine-Gray models.

Number needed to treat calculations were performed using a cross-tabulation of consent status and death on the waitlist. We used standard HIV transmission risks quoted during the informed consent process (1/20,000 for standard donor and 1/1000 for increased risk donors) to comment on the effect of consent on the risk of death relative to the risk of disease transmission.

A Cochran-Armitage test for trend was used to test for changes in consent patterns over time.

Hypothesis tests were two-sided, and a p-value less than 0.05 was considered statistically significant. Data were analysed using SAS (Version 9.4, SAS Institute Inc., Cary, North Carolina) and Stata (Version 16.1.829, StataCorp, College Station, Texas).

## Results

### Patient baseline characteristics

**Fig. 1:**
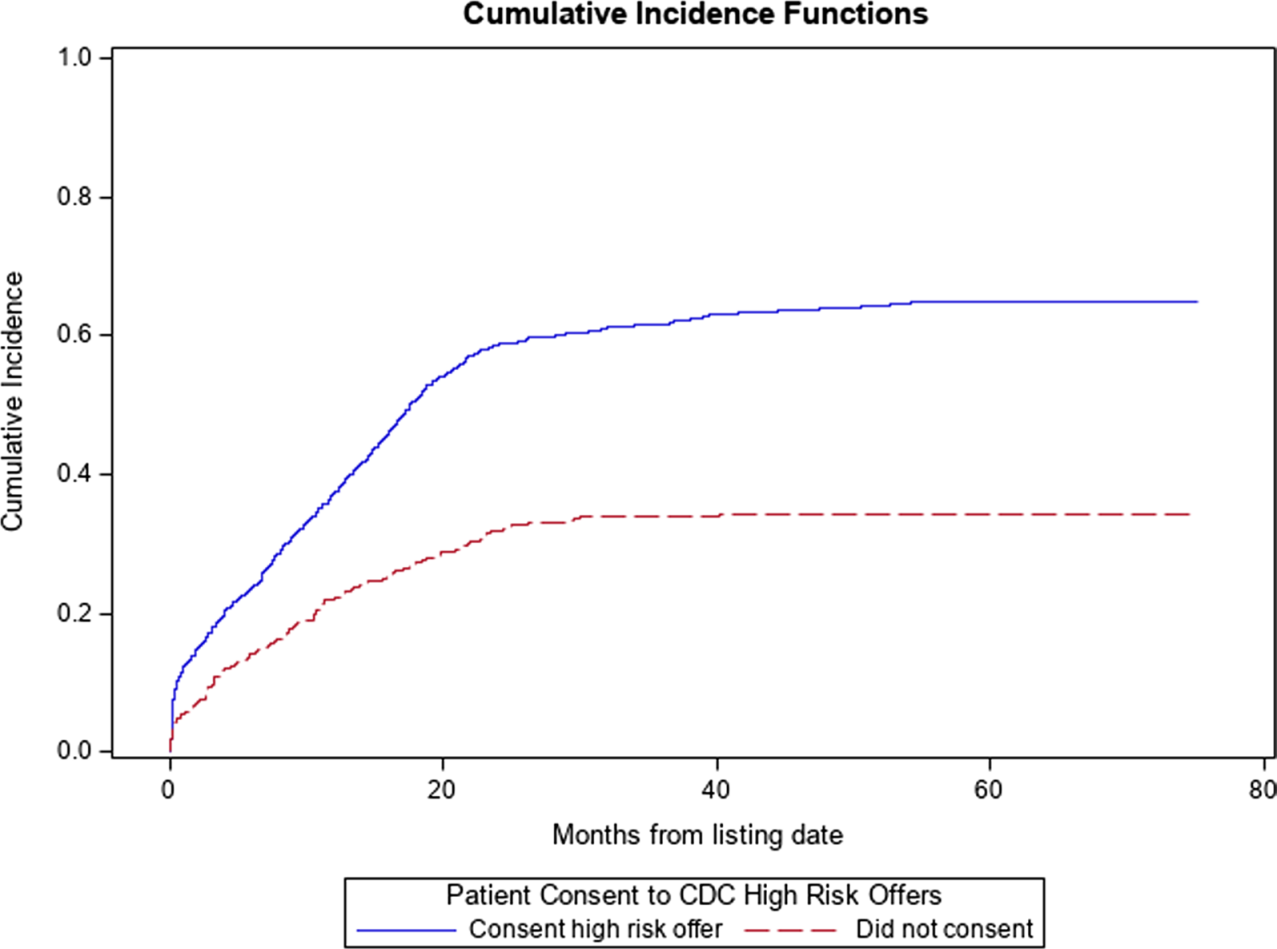
Plot of the cumulative incidence function (CIF) for the event of transplant with competing risk events of death and removal from the waitlist for other reason, stratified by consent to PHS-IR offer status

**Fig. 2:**
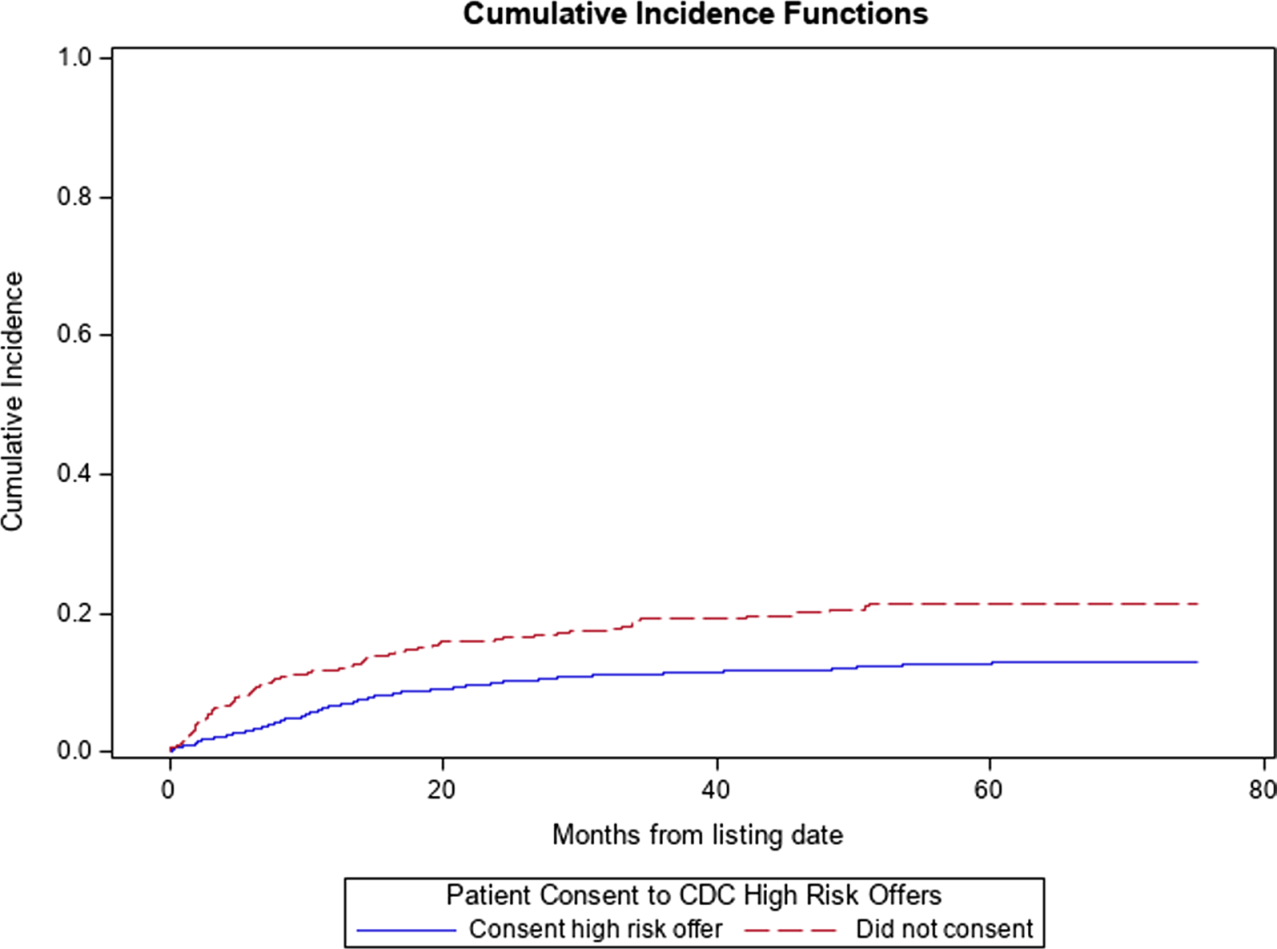
Plot of the cumulative incidence function (CIF) for the event of death with competing risk events transplant and removal from the waitlist for other reason, stratified by consent to PHS-IR offer status

A total of 1603 patients were included in our analysis. Of the patients included in the analysis, 1244 (77.6%) consented to receiving organ offers from PHS-IR donors, whereas 359 (22.4%) did not consent to offers from these donors. Baseline characteristics between patients who did and did not consent to organ offers from PHS-IR donors did not significantly differ in terms of gender, race, blood type, height, or creatinine (Table 1). MELD at listing did differ significantly between groups, with those consenting to offers from PHS-IR donors having an average MELD of 18 ± 8.9 versus 15 ± 7.2 in those who did not consent (*p* < 0.001). They also differed significantly in age at listing (56 ± 12 versus 54 ± 15 years, *p* = 0.03), total bilirubin at listing (5.8 ± 8.5 versus 4.2 ± 6.8 mg/dl, *p* < 0.001), INR at listing (1.7 ± 0.8 versus 1.5 ± 0.6, *p* < 0.001), weight (84 ± 23 versus 79 ± 24 kg, *p* < 0.001), and BMI (28 ± 8.5 versus 26 ± 9 kg/m^2^, *p* < 0.001) (Figs. 1 and 2).

**Table 1:** Patient Demographics by Consent Group

		Consent to PHS-IR Offer		*p* value
		No N = 359	Yes N = 1244	
Age at Listing		54 (15)	56 (12)	0.03
Gender	Female	141 (39.3%)	453 (36.4%)	0.32
	Male	218 (60.7%)	791 (63.6%)	
Race	Asian	64 (17.9%)	173 (13.9%)	0.097
	Black	17 ( 4.8%)	59 ( 4.7%)	
	Hispanic/Latino	92 (25.8%)	384 (30.9%)	
	Native American/ Pacific Islander	13 ( 3.6%)	27 ( 2.2%)	
	White	171 (47.9%)	601 (48.3%)	
	[missing]	[2]	[0]	
Blood Type	A	123 (34.5%)	431 (34.6%)	0.98
	AB	16 ( 4.5%)	55 ( 4.4%)	
	B	51 (14.3%)	167 (13.4%)	
	O	167 (46.8%)	591 (47.5%)	
	[missing]	[2]	[0]	
MELD at Listing		15 (7.2)	18 (8.9)	< 0.001
	[missing]	[4]	[0]	
Total Bilirubin at Listing (mg/dl)		4.2 (6.8)	5.8 (8.5)	< 0.001
	[missing]	[0]	[1]	
INR at Listing		1.5 (0.6)	1.7 (0.8)	< 0.001
	[missing]	[0]	[2]	
Creatinine at Listing (mg/dl)		1.2 (1.2)	1.3 (1.4)	0.20
	[missing]	[10]	[5]	
Height (m)		1.6 (0.26)	1.7 (0.25)	0.46
	[missing]	[3]	[18]	
Weight (kg)		79 (24)	84 (23)	< 0.001
	[missing]	[24]	[72]	
BMI (kg/m^2^)		26 (9)	28 (8.5)	< 0.001
	[missing]	[24]	[72]	
Received Increased Risk Organ at Transplant	No	63 (100.0%)	432 (70.2%)	< 0.001
	Yes	0 ( 0.0%)	183 (29.8%)	
Native MELD at Transplant		17.8 (10)	22.4 (10.7)	< 0.001
	[missing]	[8]	[7]	
Match MELD at Transplant		32 (22)	34 (13)	0.345
	[missing]	[26]	[52]	

Patient Demographics by Consent Group. Data are presented as mean (SD) for continuous measures, and n (%) for categorical measures. Also included are number of missing data fields for each variable

Of those who did not consent to offers from PHS-IR donors, 63/359 (17.5%) received a transplant during the study period, all of which were from standard criteria donors. Of those who did consent to offers from PHS-IR donors, 615/1244 (49.4%) received a transplant during the study period, of which 183/615 (29.8%) were from PHS-IR donors.

The native MELD at transplant for those who did consent to PHS-IR offers was 22.4 ± 10.7 compared to 17.8 ± 10 for those who did not consent (*p* < 0.001). The match MELD, which incorporates exception points, was 34 ± 13 versus 32 ± 22 for these groups respectively (*p* = 0.345).

Of the patients who did consent to receiving PHS-IR donor offers, 34% had at least 1 living donor candidate, comparted to 26% of those who did not consent to PHS-IR offers (p = 0.004). Of the patients who went on to receive a liver transplant, 9% of patients who had consented to receiving offers from PHS-IR donors actually received a transplant from a living donor, comparted to 33% of those did not consent to offers from PHS-IR donors (p = 0.0001).

### Time to transplant

Compared to those who did not consent to PHS-IR offers, those who did consent had 2.3 times the rate of transplant (SHR 2.29, 95% CI 1.88–2.79, *p* < 0.0001), with a median time to transplant of 11 months versus 14 months (*p* < 0.0001) (Fig. 1). After controlling for recipient age at listing, race, gender, blood type, and MELD at listing, compared to those who did not consent to PHS-IR offers, those who did consent had 2.2 times the rate of transplant (SHR 2.20, 95% CI 1.77–2.72, *p* < 0.0001).

### Waitlist mortality

Compared to those who did not consent to PHS-IR offers, those who did consent had a 44% decrease in the rate of death (SHR 0.56, 95% CI 0.42–0.74, *p* < 0.0001) (Fig. 2). After controlling for recipient age at listing, race, gender, blood type, and MELD at listing, compared to those who did not consent to PHS-IR offers, those who did consent had a 50% decrease in the rate of death (SHR 0.50, 95% CI 0.37–0.67, *p* < 0.0001).

### Removal from the waitlist

A total of 194 patients were removed for reasons other than death or transplant (of these, 63 did not consent), with 138 of those because candidate condition deteriorated (of these 48 did not consent).

Compared to those who did not consent to PHS-IR offers, those who did consent had a 41% decrease in the rate of removal for other reasons (SHR 0.59, 95% CI 0.44–0.79, *p* = 0.0005). After controlling for recipient age at listing, race, gender, blood type, and MELD at listing, compared to those who did not consent to PHS-IR offers, those who did consent had a 40% decrease in the rate of removal for other reasons (SHR 0.60, 95% CI 0.44–0.82, *p* = 0.001).

### Changes in consent patterns over time

Looking at the rate of consenting yes to organ offers from increased risk donors over time, there was a significant positive trend over the study period, with 69.1% of patients in 2013 consenting to offers from the PHS-IR group, increased to 93.1% in 2019, *p* < 0.0001 (Table 2).

**Table 2:** Consent to Increased Risk Organ Offers by Year

Year	Consent Status		Total
	No (n = 359)	Yes (n = 1244)	
2013	42 (30.9%)	94 (69.1%)	136
2014	98 (30.1%)	227 (69.9%)	325
2015	87 (28.5%)	218 (71.5%)	305
2016	54 (21.3%)	200 (78.7%)	254
2017	44 (17.5%)	208 (82.5%)	252
2018	26 (12.1%)	189 (87.9%)	215
2019	8 (6.9%)	108 (93.1%)	116

Proportion of patients who did and did not consent to receiving organ offers from increased risk donors by year. Reported as n (%)

### Utilization of PHS-IR organs

Of those who did not consent to offers from PHS-IR donors, 63/359 (17.5%) received an organ transplant. None of these patients received an organ from a PHS-IR donor. Of those who did consent to offers from PHS-IR donors, 615/1244 (49.4%) received and organ transplant. Of these, 183/615 (29.8%) were from a PHS-IR donor.

### Number needed to treat

Using a cross tabulation of consent status and death on the waitlist, among those who did not consent to increased risk organ offers, 19.78% died and among those who did consent to increased risk offers, 11.09% died. The absolute risk difference between the groups is 0.0869 with a number needed to treat of 11.51, i.e., 12 people are needed to consent in order to prevent one death.

In the literature, the risk of window period infection of HIV, HBV, and HCV ranges from 0.03 to 32.4/10,000 in patients the various PHS-IR groups (7, 10, 11). Using a stringent viral transmission rate of 1/1000 for increased risk organs, and an increased risk organ utilization rate of 30% for those consenting to increased risk organs, consenting 3334 individuals (1000/0.3) would result in 1 viral transmission and would prevent 277 deaths (3334/12).

## Discussion

We hypothesized that patients who consented to receive organ donation offers from PHS-IR designated donors would have a decreased time to transplant and a lower waitlist mortality compared to patients who did not consent to receiving organ offers from PHS-IR donors. Our study supports this hypothesis and shows that patients who consent to organ donation offers from PHS-IR donors do indeed have significantly increased rate of transplant, decreased waitlist mortality rate, and decreased waitlist removal for reasons other than transplant. In our cohort, we found that even after controlling for recipient age at listing, race, gender, blood type, and MELD at listing, acceptance of offers PHS-IR donors still led to the same findings.

While previous studies have shown that organs from PHS-IR donors are declined at higher rates than those from standard donors when offered, there is a paucity of literature on recipient consent to receiving offers from PHS-IR donors and its effects on waitlist outcomes (23–25). We expected a much lower rate of consent to PHS-IR offers, instead finding that 1244 out of 1603 (77.6%) patients did in fact consent to receiving PHS-IR organ offers.

In fact, looking at the rate of consenting yes annually over the study period, there was a significant increase, with 69.1% of patients in 2013 consenting to offers from the PHS-IR group, increased to 93.1% in 2019. This increase could reflect increased understanding of the safety profile of these organs among our hepatologists, who consent perform the informed consent with our patients at their clinic evaluation. We were also surprised to see that of those consenting to receiving these offers who were transplanted, 183/616 (29.8%) patients actually received a PHS-IR organ. Overall, these numbers reflect a rate of acceptance and utilization of these organs which seem to portend a favorable outcome for patients on the liver transplant waiting list. With greater utilization of PHS-IR organs, such that a larger proportion of consenting patients actually receive these underutilized organs, we would expect even lower mortality and time to transplant.

In calculating a number needed to treat, we found that consenting 12 patients to receive organ offers from PHS-IR donors prevents one death on the waitlist. When comparing this benefit of reduced mortality to the risk of disease transmission, we chose a stringent rate of infectious disease transmission for PHS-IR organs (1/1000). As stated previously, the literature shows the risk of window period infection of HIV, HBV, and HCV ranges from 0.03–32.4/10,000 in patients the various PHS-IR (7, 10, 11). Using these numbers, along with a 30% rate of actually receiving an increased risk organ in our cohort, we can state that for every 3334 patients consented for a PHS-IR offer, 277 deaths are prevented and one viral transmission occurs. We believe that the risk of transmission of HIV maybe even lower as there has not been transmission of HIV from a deceased donor since 2007 while there have been over 200,000 organ donors in this time period (3).

These data support the recent changes which have been made to PHS guidelines in 2020 and subsequently adopted by OPTN. As part of these changes, the “increased risk” terminology has been eliminated from the process of discussing organ offers with potential recipients. Additionally, the formalized informed consent process for offers from what were previously known as “increased risk” donors has been eliminated. Now potential recipients are informed of a donor’s individualized risk profile at the time of organ offer and a standardized informed consent is no longer required.

Our findings and the PHS 2020 policy change raise questions about the informed consent process in general, in particular the mandate for an informed consent when the risks are very low and the adverse consequences of refusing the risk, death and delay of transplantation, are orders of magnitudes higher. The institution of the original PHS policy was driven by the publicity around HIV transmission from one donor to a number of recipients. One of the purposes of the 2013 guideline was to “enhance informed decision-making by transplant candidates and families” (22). Our data suggests that this enhanced informed decision-making would lead to 277 people dying to prevent the transmission of 1 viral infection. While an “inadequate” consent process, resulting in patients refusing to accept organs because the risk versus benefit were not explained carefully enough, could be blamed for this outcome, this begs the question of whether a reasonable person needs to know the donor’s risk status if an “adequate” consent process would lead to acceptance of offers from increased risk donors. In this context, it is interesting that the CDC in their 2020 update stated they “support the development and use of tools and processes to educate transplant providers and enhance the process of transplant candidate counseling to increase organ use.” This suggests that the optimal consent process would result in all recipients accepting the increased risk donor organs.

The 2020 CDC recommendations still ask transplant centers to have consent discussions with recipients prior to transplantation of organs from increased risk designated donors and to discuss that the risk of contracting HIV, HCV, and/or HBV from the donor is “low but not zero”. The question then is how much greater than zero does the risk need to be in order for it to be part of the consent process. Risk perception research has demonstrated that public perception of risk and individuals’ anxieties around certain risks are linked to both skewed media coverage as well as strong emotions reactions to certain risks which are difficult to rationalize away (26, 27). This can lead some patients to make decisions that lead to a paradoxical increased risk of death or adverse outcome, decisions which expert consensus would say a rational person would not make. In our case, the CDC breakpoint for disclosing risk of viral transmission seems to be somewhere between the risk of transmission from a standard donor (1/10,000) and the risk of transmission from an “increased risk” (1/1000).

We recognize the potential reputational, legal, and financial risks to the CDC and transplant centers if there was a HIV transmission and the patient was not informed of the increased risk from the donor. We suspect that it is this institutional risk that leads to the dichotomy of having a policy mandating this risk disclosure to the patient but also calling upon transplant centers to “enhance the process of transplant candidate counseling to increase organ use”.

These issues represent a conflict between the principle of autonomy, where the potential recipient is informed of all risks, and the principles of non-maleficence and beneficence, where the physician is causing harm by disclosing risks that are very small relative to the benefits and leading to decreased acceptance and utilization of a scare resource.

Our study is limited by various factors. Firstly, it is a retrospective analysis which has been performed from single-center data over a limited time period, due to the relatively recent introduction of the updated PHS-IR designation. Additionally, due to data availability and collection, our ability to control for various patient demographics and covariates was limited. A propensity score-matched model may have alleviated potential issues with baseline demographic differences between PHS-IR strata, but would not have been feasible to perform with our limited sample size and available demographics.

## Conclusions

Decisions by patients to decline previously designated “increased risk” organ offers maybe driven more by fear elicited by the words “HIV”, “hepatitis B”, and “hepatitis C” rather than a rational appraisal of the risk of contracting these infections compared to the risk of dying on the waitlist. This dilemma is an interesting contrast between providing the patient with information about risks, patient autonomy, and the principle "primum non nocere", or first do no harm. As has been previously demonstrated, the risk of clinically significant communicable disease is minimal, with no appreciable detrimental outcomes for transplant recipients. This combined with our findings of increased rate of transplant, decreased mortality rate, and decreased waitlist removal in patients accepting PHS-IR organ offers, suggest that widespread utilization of these organs may offer improved patient outcomes at minimal risk. The question remains whether the risk of viral transmission by the donor organ warrants discussion with the recipient.

## Data Availability

The data that support the findings of this study are available on request from the corresponding author. The data are not publicly available due to privacy or ethical restrictions but are available from the corresponding author on reasonable request.

## Abbreviations

BMI: Body Mass Index
CDC: Centers for Disease Control
CIF: Cumulative incidence function
HBV: Hepatitis B virus
HCV: Hepatitis C virus
HIV: Human immunodeficiency virus
MELD: Model for End-stage liver disease
OPTN: Organ procurement and transplantation network
PHS-IR: Public Health Service-Increased Risk
